# P-680. Estimated Respiratory Syncytial Virus (RSV)-Related Hospitalizations and Deaths among Adults in Norway between 2010–2019

**DOI:** 10.1093/ofid/ofae631.876

**Published:** 2025-01-29

**Authors:** Maribel Casas, Tor Molden, Caihua Liang, Robin Bruyndonckx, Worku Biyadgie Ewnetu, Pimnara Peerawaranun, Solomon Molalign Moges, Aleksandra Polkowska-Kramek, Bradford D Gessner, Elizabeth Begier

**Affiliations:** P95, Terrassa, Catalonia, Spain; Pfizer, Oslo, Oslo, Norway; Pfizer Inc, New York, New York; P95, Terrassa, Catalonia, Spain; P95, Terrassa, Catalonia, Spain; P95, Terrassa, Catalonia, Spain; P-95, KESSEL-LO (LEUVEN), Vlaams-Brabant, Belgium; P95, Terrassa, Catalonia, Spain; Pfizer Biopharma Group, Collegeville, Pennsylvania; Pfizer Vaccines, Dublin, Dublin, Ireland

## Abstract

**Background:**

RSV can trigger acute cardiac events and cause respiratory disease in adults, but the true burden of RSV-associated disease and mortality remains largely underestimated due to limited standard-of-care testing and reduced test sensitivity. We retrospectively estimated the incidence of hospitalizations and deaths attributable to RSV in adults in Norway from 2010 to 2019

**Figure:**
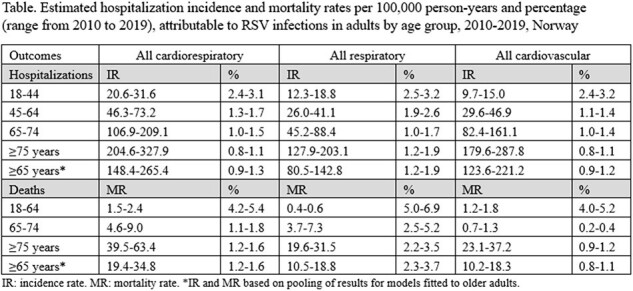

**Methods:**

Counts of weekly hospitalizations and monthly deaths due to cardiorespiratory (ICD-10 codes: I00-I99, J00-J99), respiratory (J00-J99), and cardiovascular (I00-I99) diseases were obtained from the Norwegian Patient Registry and Norwegian Cause of Death Registry national databases, respectively. A quasi-Poisson regression model was fitted, stratified by age group, to estimate the number of hospitalizations and deaths attributable to RSV while considering temporal trends and virus activity. RSV and influenza hospitalizations in children aged < 2 years and adults aged ≥65 years, respectively, were used as proxies for viral activity. Annual RSV-attributable hospitalization incidence and mortality rates were calculated using populations at risk as denominator.

**Results:**

For all three study outcomes, RSV-attributable incidence and mortality rates increased with age. The highest RSV-attributable rates were observed in patients aged ≥65 years (range from 2010 to 2019 - hospitalizations per 100,000 person-years: 148-265 for cardiorespiratory, 81-143 for respiratory, and 124-221 for cardiovascular; deaths per 100,000 person-years: 19-35 for cardiorespiratory, 11-19 for respiratory, and 10-18 for cardiovascular). In any given year, the RSV-attributable cardiorespiratory disease hospitalization incidence and mortality rates were two times higher than the rates for respiratory disease alone. For the three outcomes, the attributable proportion of RSV hospitalizations was 2 to 3 times higher in adults aged 18-44 years than in those aged ≥65 years.

**Conclusion:**

In Norway, RSV contributes substantially to both respiratory and cardiovascular diseases across all adult age groups, emphasizing the need for effective preventive strategies. Including cardiovascular disease increased measured burden by nearly 2-fold compared to respiratory disease alone.

**Disclosures:**

**Maribel Casas, PhD**, Pfizer: Advisor/Consultant **Tor Molden, PhD**, Pfizer: I am an employee of Pfizer **Caihua Liang, MD, PhD**, Pfizer: Stocks/Bonds (Private Company) **Aleksandra Polkowska-Kramek, n/a**, Pfizer: employee of P95 which received funding from Pfizer to conduct this study **Bradford D. Gessner, M.D., M.P.H.**, Pfizer: Employee|Pfizer: Stocks/Bonds (Public Company) **Elizabeth Begier, MD, M.P.H.**, Pfizer Vaccines: Employee|Pfizer Vaccines: Stocks/Bonds (Private Company)

